# Effects of electric charge on fracture healing

**DOI:** 10.1038/s41598-022-20153-3

**Published:** 2022-09-23

**Authors:** Ling He, Yingling Yao, Nan Wang, Guoxin Nan

**Affiliations:** 1 Department of Orthopaedics Children’s Hospital of Chongqing Medical University, National Clinical Research Center for Child Health and Disorders, Ministry of Education Key Laboratory of Child Development and Disorders, Chongqing, 400014 China; 2 Chongqing Engineering Research Center of Stem Cell Therapy, Chongqing, China; 3 Present Address: Department of Orthopaedics, Children’s Hospital of Chongqing Medical University, Chongqing, China

**Keywords:** Mechanisms of disease, Trauma

## Abstract

Fracture nonunion is a common and challenging complication. Although direct current stimulation has been suggested to promote fracture healing, differences in cell density near the positive and negative electrodes have been reported during direct current stimulation. This study aimed to explore the effects of these differences on osteoblast proliferation and fracture healing. MC3T3-E1 cells were stimulated by positive and negative charges to observe cell proliferation, apoptosis, and osteogenic factor expression in vitro, while positive and negative charges were connected to the Kirschner wires of the fractures in an in vivo double-toe fracture model in New Zealand white rabbits and fracture healing was assessed in digital radiography (DR) examinations performed on days 1, 15, 30. Bone tissue samples of all rabbits were analysed histologically after the last examination. The results showed that in comparison with the control group, after DC stimulation, the number of cells near the positive electrode decreased significantly (P < 0.05), apoptosis increased (P < 0.05), the expression of osteocalcin, osteoblast-specific genes, and osteonectin decreased significantly near the positive electrode (P < 0.05) and increased significantly at the negative electrode (P < 0.05). The fracture at the positive electrode junction of New Zealand white rabbits did not heal. Histomorphological analysis showed more bone trabeculae and calcified bone in the bone tissue sections of the control group and the negative electrode group than in the positive electrode group. The bone trabeculae were thick and showed good connections. However, positive charge inhibited osteoblast proliferation and a positive charge at fracture sites did not favour fracture healing. Thus, a positive charge near the fracture site may be a reason for fracture nonunion.

## Introduction

Fracture nonunion is a common and challenging complication in the treatment of fractures. About 5–10% of long bone fractures may show nonunion or delayed union^1^. At present, there is no uniform definition of fracture nonunion. The U.S. Food and Drug Administration (FDA) defines fracture nonunion as incomplete healing within 9 months after fracture and no progressive signs of healing on radiographs for three consecutive months^2^. Fracture nonunion causes long-term pain, physical disability, mental health problems, and continuing medical expenses, which have a major impact on patients' life^3^.

The process of fracture healing involves many endogenous and exogenous factors, and disruption of these factors may lead to delayed union or nonunion^4,5^. Many risk factors are considered to cause nonunion, including patient-dependent factors such as age, sex, medical comorbidities, medications, and nutritional status as well as patient-independent factors such as fracture type and characteristics, infection, fracture fixation implants, and iatrogenic factors^6–8^. Although many studies have evaluated the causes of fracture nonunion and substantial progress has been made in the treatment of nonunion, nonunion can occur even in stable fractures without any of these risk factors. A retrospective study showed that multiple fractures are more likely to cause nonunion: while the incidence of nonunion in patients with one fracture was 4.4%, the incidence increased with the number of fractures and was 24% in patients with seven or more fractures^9^. In addition to the influencing factors we currently know of, there may be other unique factors influencing nonunion.

Bone tissue repair and reconstruction involve complex biological and biomechanical mechanisms mediated by the effects of bone marrow mesenchymal stem cells, osteoblasts, osteoclasts, and other cells in the impaired area. High-intensity mechanical strain stimulation and endogenous current play important roles in bone growth^10^. The epiphyseal region of the long bones is electronegative, and the shaft is electroneutral. When a fracture occurs, the epiphyseal region becomes more negative and the fracture site also shows a negative charge, and this electronegative change is maintained throughout fracture healing^11^. The electronegativity trend and the secretion of cytokines after fracture affect cell proliferation, migration, differentiation, and osteogenesis^12^. Thus, will an electropositive fracture site environment induce changes in these cellular activities? To address this question, the present study aimed to observe the effects of positive and negative charges on osteoblasts as well as the effects of positive and negative charges of the same voltage and current on fracture healing.

## Methods

### Cell culture

MC3T3-E1 murine preosteoblastic cell lines obtained from Orthopaedic laboratory of Army Medical University were cultured in alpha modified Eagle medium (α-MEM, Hyclone, USA) containing 10% foetal bovine serum (FBS; Gicbo, USA) and 1% antibiotic and antimycotic solution. Cells were incubated at 37 °C, 5% CO_2_, and atmospheric O_2_. In the experiments, cells were seeded into six-well plates at a density of 300,000 cells per well, and the media volume was kept at 3 mL. Media change was performed on the following day after seeding before applying electrical stimulation.

### DC-stabilised electrostimulation system setup

**Figure 1:**
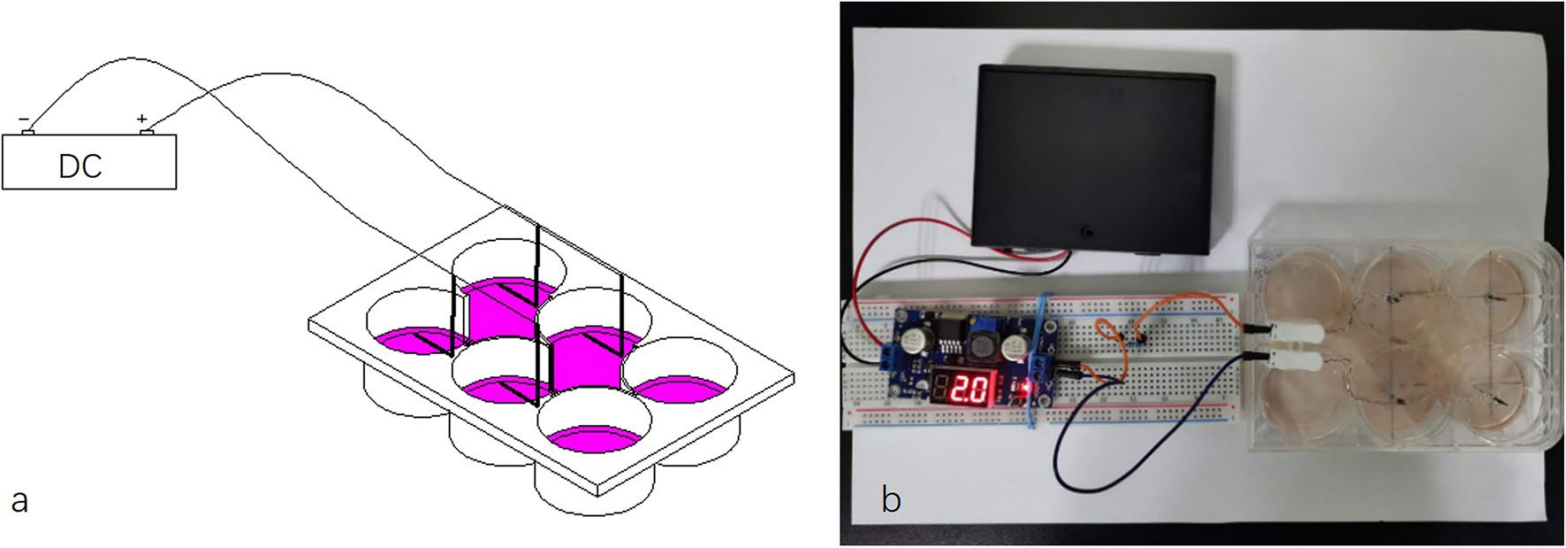
EStim cell culture chamber.

The DC stimulation chamber was used in this study was improved based on the original design described by Mobini and Leppik et al.^13^. The current output terminal was powered by six 1.5 V batteries. The circuit integrates an adjustable buck regulator power supply module (LM2596S) to maintain a constant output voltage and increase or decrease the resistance component through the required current. The current output terminal connects a wiring terminal fixed on the side of the 6-hole cover. The middle line of each hole was drilled on the 6-hole plate cover such that the distance between the two holes was 30 mm. A titanium alloy wire (0.8 mm in diameter) was used as the implanted electrode, and the effective length of the implanted electrode was 20 mm. The electrode was paralleled with a silver-coated copper cable, and the loose end of the copper wire was connected to the wiring end on the left side of the 6-hole plate cover. The six-hole plate for cell inoculation was processed to communicate between the parallel holes. The schematic and physical drawings of the device are shown in Fig. 1.

### Cell proliferation

MC3T3‐E1 cells were stimulated with direct current for 24 and 48 h. A control group of cells cultured without electrical stimulation was also maintained. The cells at the anode and cathode and those of the control group were collected separately; the respective cell suspensions were mixed with 0.4% trypan blue (catalog# 15250061; Gicbo, USA) in a 9:1 ratio, and 20 µL of the suspensions were added to a Countstar counting plate and observed under a microscope. The dead cells were stained blue, while the live cells were colourless and transparent, and the number of live cells were counted within 3 min.

### Apoptosis

After 24 and 48 h of electrical stimulation, cells of each group were collected separately and the cell concentration was adjusted to 1 × 10^6^/mL. In accordance with the procedure for the apoptosis assay kit (BD Biosciences, USA), the cells were resuspended in 5-mL flow tubes; 5 µL of fluorescein isothiocyanate (FITC)-Annexin V and 5 µL of propidium iodide (PI) staining solution were added to the flow tubes; the tubes were mixed gently and incubated for 15 min at room temperature and protected from light; and 500 µL of 1× binding buffer was added. The FITC fluorescence level in the FITC channel and the PI fluorescence level in the PI channel were detected immediately using the method recommended in the kit. Data analysis was performed using FlowJo VX. Each experiment was repeated three times.

### Real-time quantitative PCR

After 7 days of cell culture, the cells at the positive and negative electrodes as well as those in the control group were collected. Total RNA of the cells was extracted by SimplyP total RNA extraction kit and reverse-transcribed into cDNA. The expression levels of osteocalcin, osterix, and osteonectin were evaluated by real-time quantitative PCR. The experiments were repeated three times, with GAPDH as the internal reference, and the relative expression levels of osteocalcin, osterix, and osteonectin were calculated by 2-∆∆Ct method. The primer sequences used were as follows (Table 1).

**Table 1:** Primers used in Real time quantitative PCR.

Primer gene	Sequence (5′–3′)
Oxterix	Forward:5′-AGGTCTGATGGGACAGAGTGA-3′
	Reverse:5′-GGGCTGAAAGGTCAGCGTAT-3′
Osteocalcin	Forward:5′-CCTTCATGTCCAAGCAGGA-3′
	Reverse:5′-GGCGGTCTTCAAGCCATAC-3′
Osteonectin	Forward:5′-GATGGGATGTTGTCCCTTCCC-3′
	Reverse:5′-GAGACACTGGGTAGTGCAGG-3′
GAPDH	Forward:5′-GGCTGCCCAGAACATCAT-3′
	Reverse:5′-CGGACACATTGGGGGTAG-3′

### Animals and fracture model

**Figure 2:**
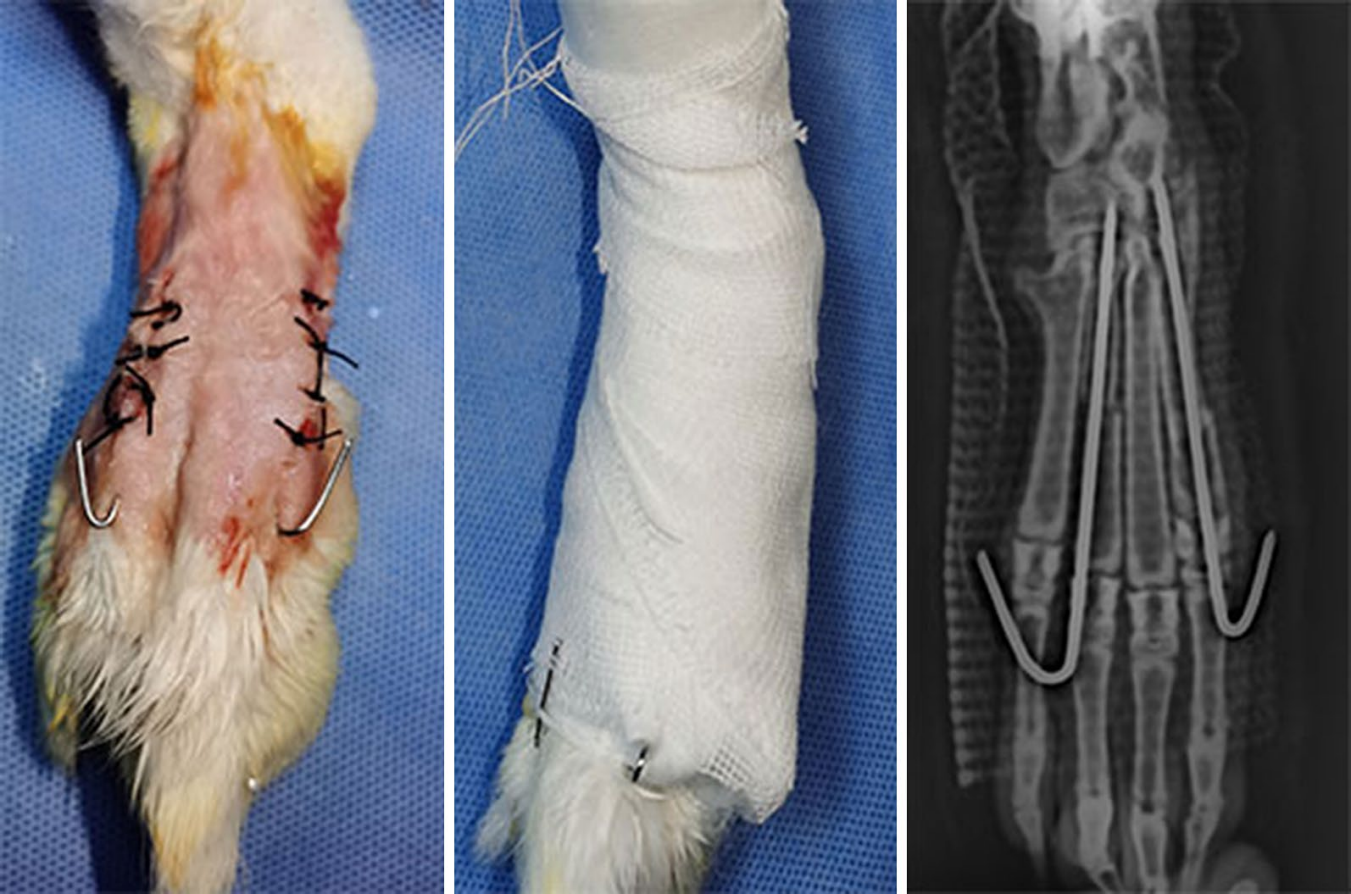
Double-fracture model of the toe bone of the New Zealand great white rabbit.

A total of 12 New Zealand white rabbits (male; weight, 2.5–3 kg) used in the experiment were provided by a commercial vendor (Animal Certificate No.: SCXK (Yu) 2021-0010). This study was conducted with the approval of the Experimental Animal Ethics Committee of Children’s Hospital of Chongqing Medical University, China (CHCMU-IACU20210114003), and carried out in accordance with the ARRIVE guidelines. All methods were performed in accordance with the revised Animals (Scientific Procedures) Act 1986 in the UK and Directive 2010/63/EU in Europe. All animals were reared at room temperature (20 °C to 25 °C), 40%–60% relative humidity, and a 12–12-h circadian cycle; food was uniformly provided by professional breeders, and water was offered ad libitum. Before the operation, the rabbits were fasted for food and water, and 3% pentobarbital sodium was injected along the ear margin vein at a dose of 1 mL/kg for anaesthesia. The rabbits were maintained supine and fixed on the operating table; the right hind foot was exposed; a sterile towel was placed; and the surgical site was disinfected with iodophor three times. The skin was cut longitudinally between the first and third phalanges with a scalpel, and the muscles and fascia were separated to expose the phalanges. The phalanges were cut off with a bone saw to completely severe them; excess bone fragments were cleaned up; the fracture site was fixed with a 0.8-mm Kirschner wire; the wound was sutured layer-by-layer with 3-0 nylon sutures; and the rabbit double-fracture model was completed with external fixation with plaster (Fig. 2). For 3 days after the operation, all rabbits were injected intramuscularly with penicillin (80,000 U) once a day to prevent infection, and the wound was monitored for signs of infection. The animals were randomly divided into experimental and control groups, with six rabbits in each group. Rabbits in the experimental group underwent DC stimulation. Thus, immediately after modelling, the negative DC electrode was connected to the first phalangeal fixed Kirschner wire, and the positive electrode was connected to the third phalangeal fixed Kirschner wire, and continuous stimulation was applied with a 10-µA DC. The DC supply device used in the experiment was the same as that in cell experiment.

### Radiological assessment

The fracture site in all rabbits was photographed at 1 day, 0.5 months, and 1 month after modelling to observe fracture healing and the position of the kerf pins, and to evaluate fracture line changes and bone scab formation after the fracture.

### Histological analysis

At month 1, the animals were euthanised. The toe bones were dissected and fixed in 4% paraformaldehyde and then decalcified in JYBL-I decalcifying solution (catelog# G2470; Solarbio). The decalcified bones were paraffin-embedded and sectioned into 5-µm slices, which was followed by HE staining and Goldner’s trichrome staining.

### Statistical analyses

The results were statistically analysed using SPSS (version 20.0) and GraphPad Prism V.8.00. Data were expressed as mean ± standard deviation and analysed using t-test and one-way analysis of variance (ANOVA), except the histological data. Stained sections were photomicrographed and histomorphometrically analysed using Aperio ImageScope v12.1.0.5029 (Leica, USA). Statistical significance was defined by p < 0.05.

## Results

### Effects of DC polarity on cell proliferation

**Figure 3:**
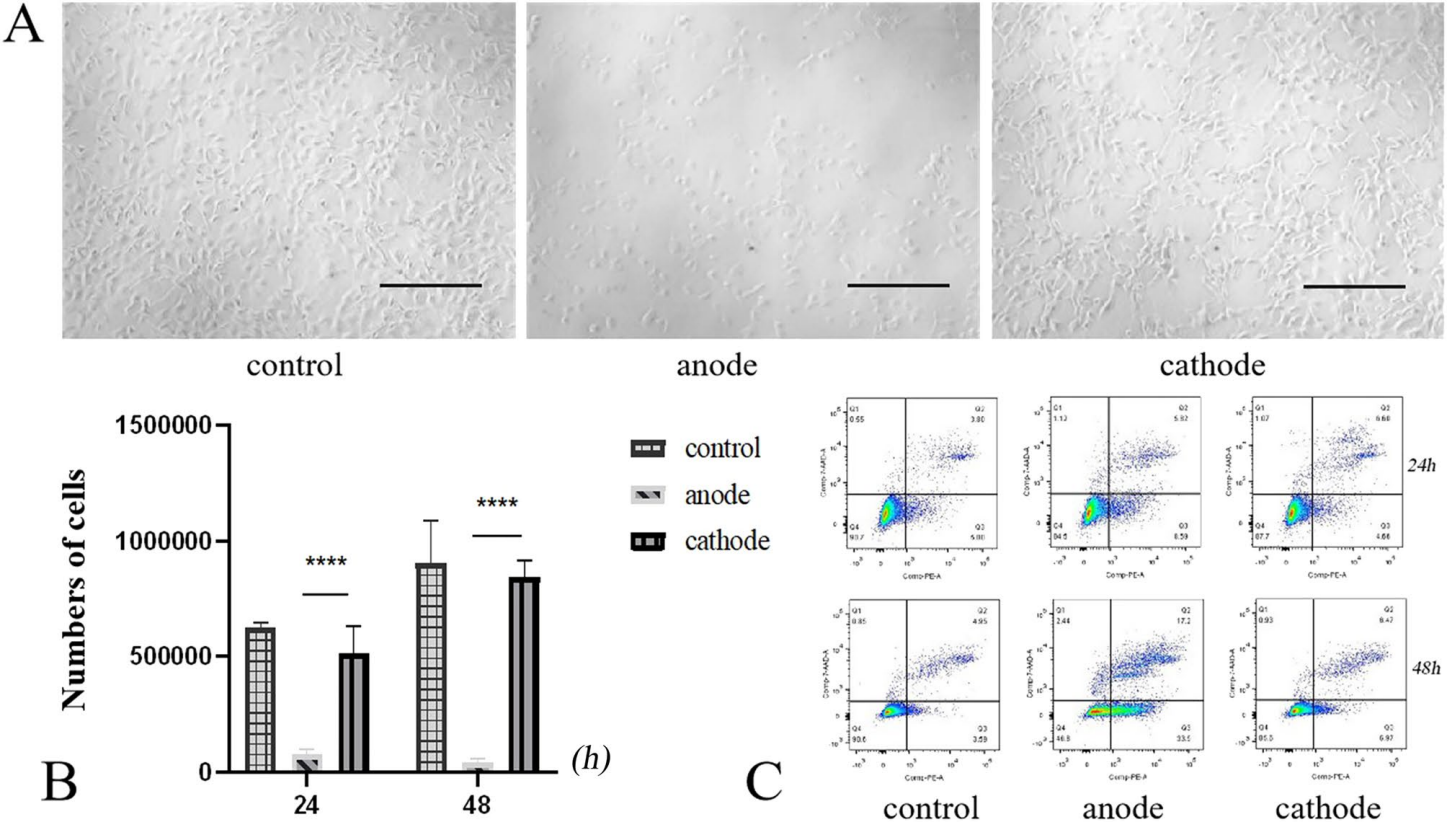
(**A**) Morphological observation of MC3T3-E1 cells under an optical microscope after two days of electrical stimulation (×100), scale bars 200 µm. (**B**) Cell counts in the positive electrode, negative electrode, and control groups after DC stimulation for 24 and 48 h. (**C**) Apoptosis of cells near the positive and negative electrodes in the control and electrical stimulation groups at 24 and 48 h.

After electrical stimulation, the cells in the control group and the negative electrode group appeared as long spindles or showed a polygonal shape, while the cells in the positive electrode group lost their original shape and became round (Fig. 3A). After 24 h, the number of MC3T3-E1 cells in the positive electrode group decreased significantly, and the growth rate of living cells was − 7.4%, while the corresponding values for the control group and negative electrode group were 107% and 71%, respectively. After 48 h of electrical stimulation, the growth rate in the positive electrode group was − 86.7%, while the corresponding values for the control electrode and negative electrode groups were 201% and 181% respectively. Thus, in comparison with the control and negative electrode groups, the positive electrode group showed a significant reduction in the number of cells (p < 0.05). However, no significant difference in cell proliferation was observed between the negative electrode and control groups (p > 0.05) (Fig. 3B).

### Effects of DC polarity on cell apoptosis

After 24 h of electrical stimulation, the apoptosis rates of cells in the positive and negative electrode groups were not significantly different from that in the control group. After 48 h of electrical stimulation, the apoptosis rate of cells in the positive electrode group was significantly higher than that in the negative electrode and control groups (P < 0.05) (Fig. 3C).

### Effects of DC polarity on osteocalcin, osterix, and osteonectin expression

**Figure 4:**
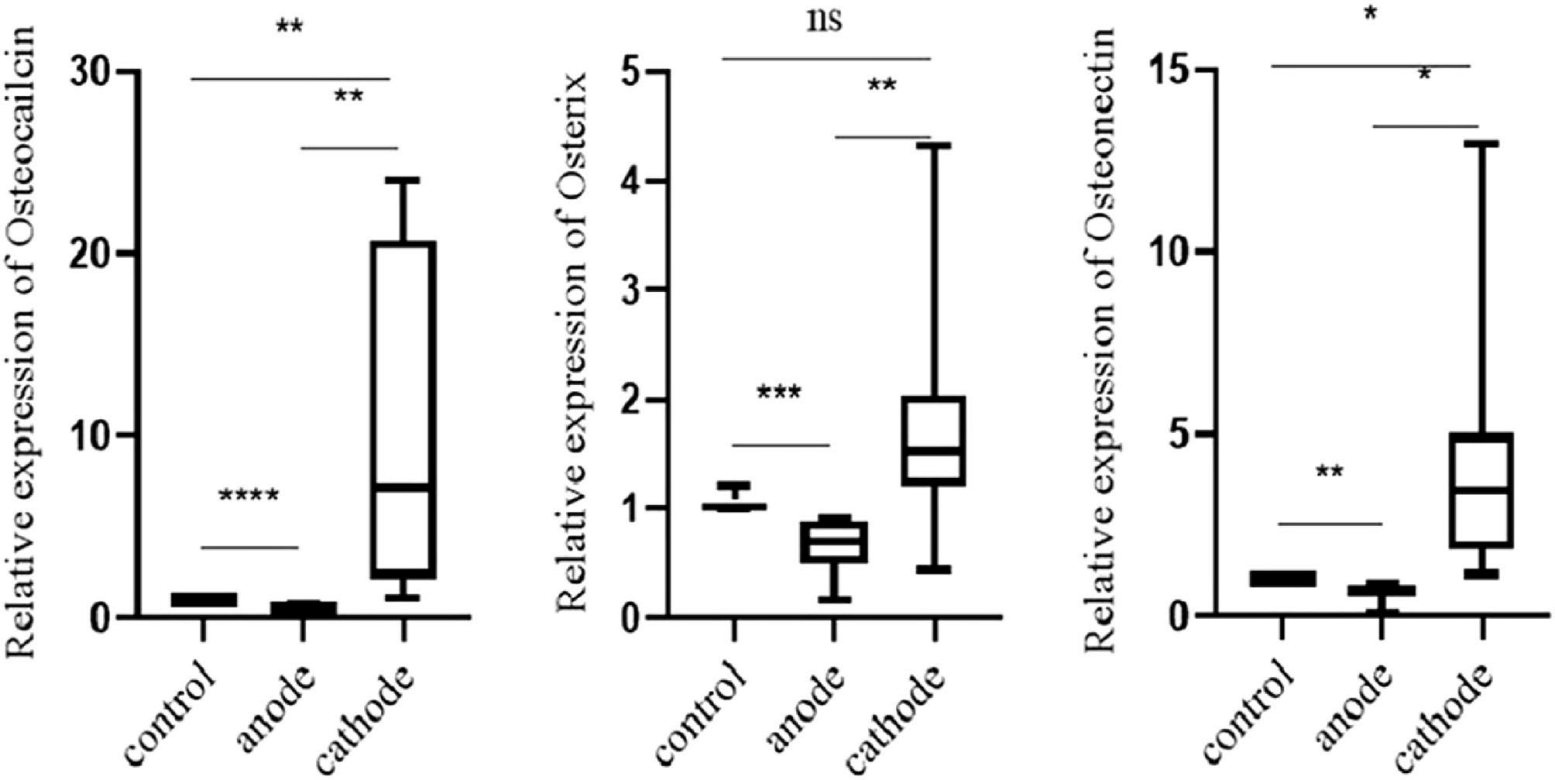
The expression levels of osteocalcin, osterix, and osteonectin in MC3T3-E1 cells of different electrode and control groups were detected by qPCR. ^ns^P > 0.05, *P < 0.05, **P < 0.01, ***P < 0.001, ****P < 0.0001.

The specific expression levels of osteocalcin, osterix, and osteonectin in MC3T3-E1 cells were quantitatively detected by real-time PCR. In comparison with the control group, the positive electrode group showed reduced expression of these genes (P < 0.05), but their expression increased significantly in the negative electrode group (P < 0.05) (Fig. 4).

### Radiological assessment

**Figure 5:**
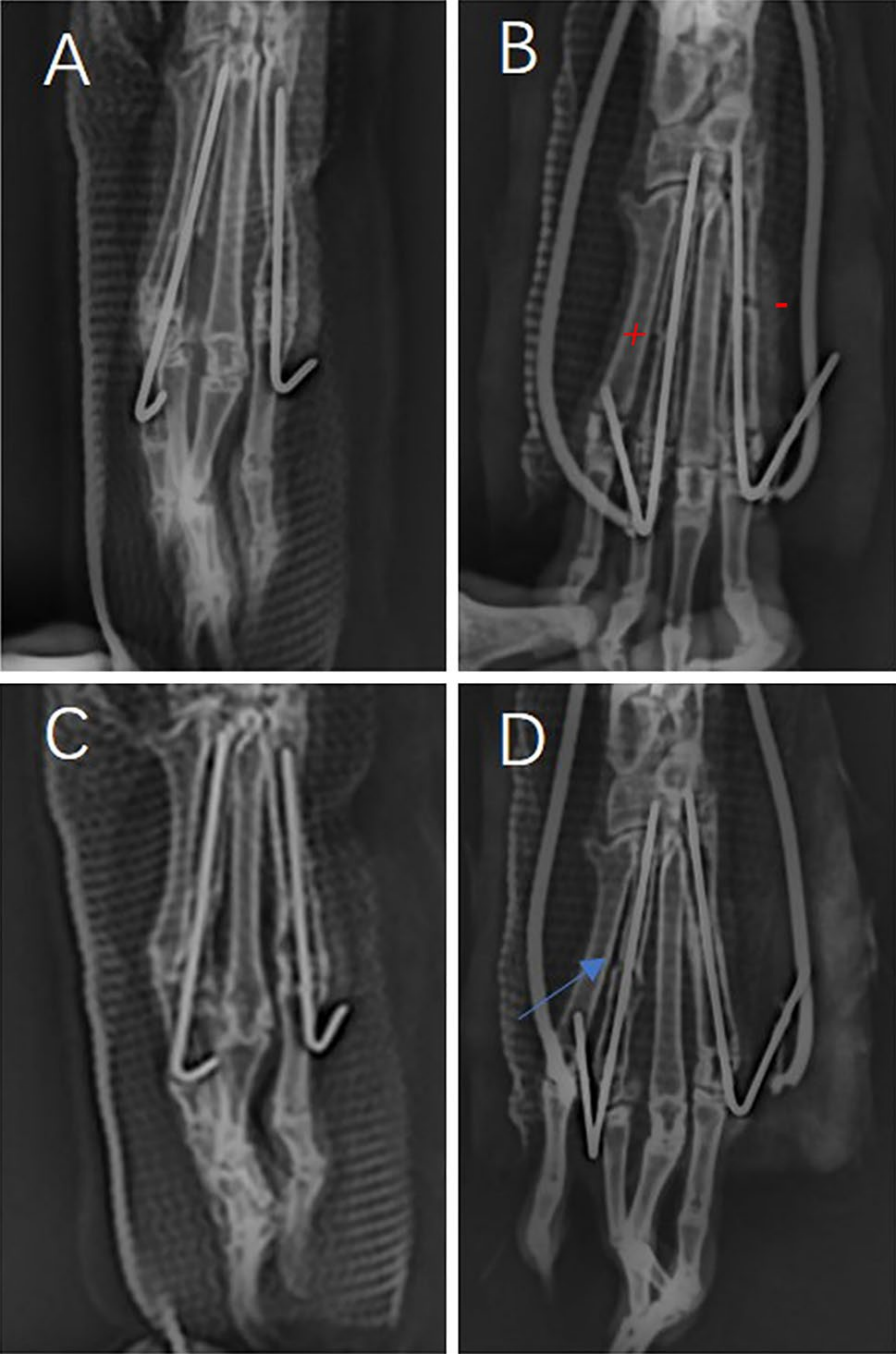
(**A**,**B**) Toe DR images one day after fracture; (**C**) toe DR image 1 month after surgery in the control group, where the fracture line had disappeared; (**D**) toe DR image 1 month after surgery in the electrical stimulation group, where the fracture line indicated by the arrow is obvious (N ≥ 3).

At one month, the fracture line at the negative electrode group had disappeared and the fracture had healed, while the fracture line in the positive electrode group was clearly visible with almost no bone scab formation, indicating poor fracture healing. In the control group, all cases showed good healing of both fractures with disappearance of the fracture line (Fig. 5).

### Histological analysis

**Figure 6:**
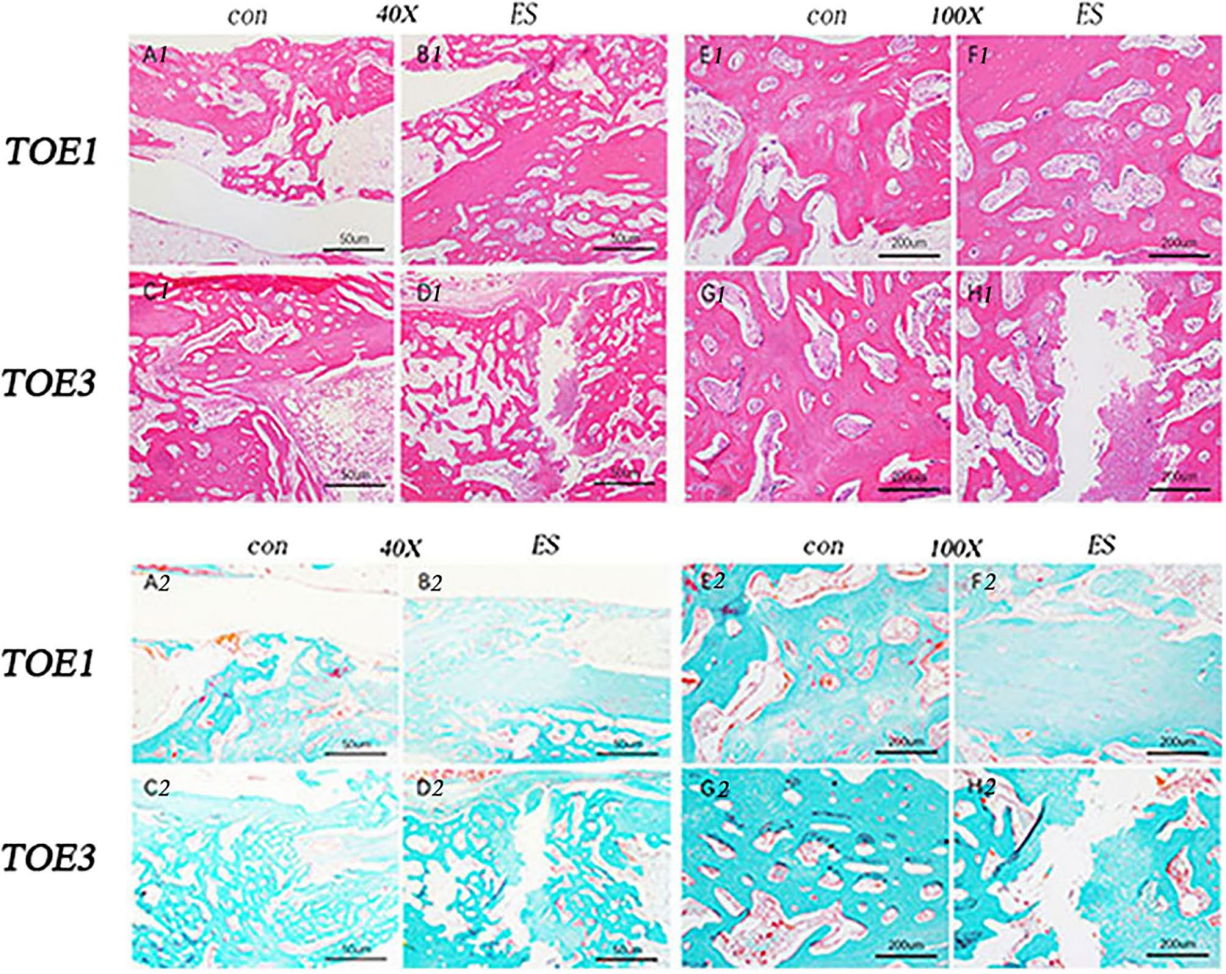
Typical tissue images stained with haematoxylin–eosin (HE) and Goldner's trichrome staining 1 month after surgery. HE staining of bone tissue in controls A1, C1, E1, and G1 and B1 and F1 at the negative electrode connection shows the formation of numerous trabeculae with no gaps between the tissues. Bone tissue D1 and H1 at the positive electrode connection shows limited trabecular formation with some areas showing neither trabeculae nor osteocytes. Goldner staining of bone tissue in controls A2, C2, E2, and G2 and at the negative electrode connections B2 and F2 shows large areas of green mineralised bone that is connected in sheets with only a small amount of bone-like material with no gap between the tissues. Bone tissue at the positive electrode junction D2 and H2 shows a large area of red osteoid at the positive electrode fracture with very little green mineralised bone (N ≥ 3).

One month after fracture, HE staining showed that a large number of osteoblasts and osteoclasts infiltrated the cartilage of the control and negative electrode groups, forming a large number of bone trabeculae. The bone trabeculae were wider and more tightly connected than those in the positive electrode group, while a large number of fibrous calluses were still formed in the positive electrode group, and no osteoblasts and bone trabeculae were found in some areas (Fig. 6). Goldner’s three-colour staining showed a large number of green mineralised bones in the control and negative electrode groups, while the positive electrode group showed a large area of red-like bone with a small amount of green mineralised bone (Fig. 6).

## Discussion

During the process of regeneration and remodelling after bone tissue injury, the changes in physiological and environmental factors will constantly reshape the bone and reconstruct bone matrix^14^. Therefore, the potential stress generated by collagen fibres in bone can promote bone reconstruction^15^. During fracture, the electronegativity of the metaphysis as well as the whole fracture site increases, and the change of electronegativity is maintained until the fracture is healed^11^.

The purpose of this study was to observe the effect of positive and negative DC electrodes on osteoblast proliferation and fracture healing in vitro. Although numerous previous studies have evaluated the effect of current on osteogenesis, the existing research mainly focused on the role of current in promoting osteogenesis, and mostly did not focus on the effects of different electrodes in the process of current stimulation^13,16,17^. The cell density near the electrode has been shown to be significantly lower than that between the electrodes, and the cell density near the positive electrode has been shown to be lower than that near the negative electrode^18^. Our in vitro study also confirmed this finding. In the same-voltage DC electric field, the proliferation of MC3T3-E1 cells near the positive electrode was significantly limited and showed a time-related accumulation effect, and apoptosis was more obvious with extension of the electrical stimulation time.

After fracture, osteoblasts proliferate into the bone marrow and eventually differentiate into bone marrow mesenchymal cells^19^. These mesenchymal stem cells and osteoblasts migrate towards the cathode due to their electrotaxis, while osteoclasts migrate towards the anode^20^. Our study mainly focused on the subsequent proliferation of osteoblasts. To better observe the proliferation of cells migrating toward the positive and negative electrodes, we improved the electrical stimulation device proposed by Mobini et al.^13^ to completely separate the cells near the positive and negative electrodes and facilitate statistical analysis. Although electrical stimulation has been mainly proven to improve the cell proliferation rate, findings indicating that electrical stimulation can reduce cell proliferation or has no effect on cell proliferation have also been reported^21^. Our results showed that in comparison with the control group, the proliferation of positive electrode cells decreased and the proliferation of negative electrode cells had no effect in the electrical stimulation group. This is related to the increase in apoptosis near the positive electrode. The mechanisms underlying inhibition of cell proliferation by electrical stimulation include cell cycle arrest, intracellular Ca^2+^ influx, and upregulation of tumour suppressor protein. Electrical stimulation activated L-type Ca^2+^ channels; at physiologically normal intracellular Ca^2+^ levels, cell proliferation and apoptosis are not affected. However, when the intracellular Ca^2+^ level crosses the threshold level for activating apoptosis, apoptosis increases^22^.

Osteocalcin, osterix, and osteonectin are important factors influencing osteoblast differentiation and bone formation, and are markers of late osteogenic differentiation^23–26^. The experimental results show that the expression of these bone-related genes in the positive electrode group was lower than that in the control group and the negative electrode group, and the expression of these genes in the negative electrode group was significantly higher than that in the control group, indicating that a positive charge reduces the expression of bone-related genes and regulates the level of osteogenesis-related pathways, which is not conducive to osteoblast osteogenesis. The negative electrode upregulates the expression of osteogenesis-related genes, which is conducive to promote the differentiation of osteoblasts into bone. The electrical field can also trigger biophysical changes on the cell surface. The function of membrane proteins is affected by changes in the charge distribution on biomolecules, such as enzyme activity (Na + /K + ATPase and Ca^2+^ ATPase), membrane receptor complex, and ion transport channel. When the current passes through the cathode, it reduces the oxygen concentration through Faraday reaction, increases the pH value, and produces hydrogen peroxide. The reduction of oxygen concentration enhances osteoblast activity^27^. The concentration of positive and negative ions in the cell culture medium was completely consistent in our experiment.

After 1 month, the fracture in the control group was completely healed, while in the experimental group, the fracture connected to the negative electrode was healed and that connected to the positive electrode was not healed. Histological results also showed that in the control group and the negative electrode group, more callus was formed, the bone trabeculae were thicker, and bone calcification was obvious, while the callus at the positive electrode junction was not completely formed. In the traditional method of DC stimulation to promote fracture healing, the speed of fracture healing differs due to differences in the placement of electrodes. A better effect can be obtained when the cathode is placed at the fracture site. However, in the traditional method, when two fractures occur in the same part, the positive and negative electrodes are connected to the two fractures simultaneously, i.e., one end of the electrode is placed at the fracture site and the other end of the electrode is in normal tissue.

In this experiment, we chose the phalange as the research site because the rabbit phalange can effectively simulate two long bones in the same part, and evaluating phalanges 1 and 3 can avoid the interaction of factors secreted by the two fracture sites during the process of fracture healing. Nevertheless, our research design still had some limitations. We regarded the time of fracture healing in the control group as the end point of the study, and did not evaluate the formation of callus at different stages in the process of fracture healing. Moreover, only one current was selected, which meant that we could not confirm the effects of different current stimuli. However, the purpose of this study was to simply detect whether positive and negative electrodes (or positive and negative charges) will affect fracture healing, and the selected current is 10 µA, which is the minimum current required for tissue healing. Our results clearly showed that positive electrodes significantly inhibit fracture healing.

## Conclusions

The findings indicate that the positive electrode (positive charge) inhibits the proliferation of osteoblasts, downregulates the expression of osteocalcin, osteoblast-specific genes, osteonectin and other osteogenic genes, and inhibits cell osteogenesis. In contrast, the negative electrode upregulates osteogenesis-related genes and promotes osteogenesis. After fracture, the fracture site with a positive charge will show delayed healing or nonunion. An abnormal charge at the fracture site may be one of the causes of fracture nonunion.

## Data Availability

The datasets used and/or analyzed during the current study available from the corresponding author on reasonable request.
